# Machine learning for classifying narrow-beam electron diffraction data

**DOI:** 10.1107/S2053273323004680

**Published:** 2023-06-20

**Authors:** Senik Matinyan, Burak Demir, Pavel Filipcik, Jan Pieter Abrahams, Eric van Genderen

**Affiliations:** a Biozentrum, University of Basel, Basel, Basel-Stadt, Switzerland; b Laboratory of Nanoscale Biology, Paul Scherrer Institute, Villigen, Switzerland; Czech Academy of Sciences, Czech Republic

**Keywords:** diffraction, single-molecule electron diffraction, TEM, transmission electron microscopy, machine learning, neural networks

## Abstract

As an alternative approach to X-ray crystallography and single-particle cryo-electron microscopy, single-molecule electron diffraction has a better signal-to-noise ratio and the potential to increase the resolution of protein models. This technology requires collection of numerous diffraction patterns, which can lead to congestion of data collection pipelines. However, only a minority of the diffraction data are useful for structure determination because the chances of hitting a protein of interest with a narrow electron beam may be small. This necessitates novel concepts for quick and accurate data selection. For this purpose, a set of machine learning algorithms for diffraction data classification has been implemented and tested. The proposed pre-processing and analysis workflow efficiently distinguished between amorphous ice and carbon support, providing proof of the principle of machine learning based identification of positions of interest. While limited in its current context, this approach exploits inherent characteristics of narrow electron beam diffraction patterns and can be extended for protein data classification and feature extraction.

## Abstract 2

Neural networks were trained for robust classification of narrow electron beam diffraction patterns and may significantly decrease the need for storage space.

## 1. Introduction

Machine learning (ML) techniques enable extraction of sophisticated features from large data sets and can generate state-of-the-art performance in a variety of biomedical applications (Zhang *et al.*, 2021 ▸). Algorithms based on deep learning architectures, which represent a custom set of non-linear input–output estimators for the given data, can surpass expert human performance and successfully model very complex phenomena (Ede, 2021 ▸).

Compared with traditional learning techniques, where most of the applied features need to be marked by a domain expert, deep learning architectures can extract the feature space in an incremental manner and provide an end-to-end ‘black box’ solution. This eliminates the need for manual feature extraction and domain expertise. However, they lack interpretability and require a large amount of training data (Bailly *et al.*, 2022 ▸). Training is computationally expensive and time-consuming due to the number of parameters. These limitations have been partially removed by better availability of high-end computers, an exponentially increased quantity of data being generated, and greater interest in the area from both the industrial and scientific sectors (Bishop, 2013 ▸).

In addition, the number of applications in transmission electron microscopy (TEM) is growing (Treder *et al.*, 2022 ▸; Yonekura *et al.*, 2021 ▸). The emergence of direct electron and hybrid pixel detectors (HPDs) (Fröjdh *et al.*, 2022 ▸; Takaba *et al.*, 2021 ▸) has changed the cryo-EM (cryo-electron microscopy) field significantly (Faruqi & McMullan, 2018 ▸). If combined with advanced data collection and analysis techniques, this could enable structural biologists to reveal the structure of complex biomolecular machines at near-atomic resolution. However, due to the generation of large amounts of data, traditional data handling strategies are becoming increasingly unwieldy. It is now possible to capture images at a relatively high frame rate compared with other types of detectors in the field, and existing data analysis pipelines struggle to keep up with the data production rate. Huge amounts of data need to be kept, and meeting the rate of data growth and the transfer rate is challenging for storage devices (Horwath *et al.*, 2020 ▸; Taheri *et al.*, 2016 ▸). ML algorithms are likely to have a drastic impact on this bottleneck.

Our group is analyzing far-field electron scattering diffraction data generated by diffracting a 10 to 60 nm narrow, parallel electron beam on a protein sample. By assuming the beam is not much wider than the size of the protein of interest, this approach is likely to improve the signal-to-noise ratio compared with cryo-EM imaging (Latychevskaia & Abrahams, 2019 ▸). HPDs have proven to be better for these types of data than the direct electron detectors used for cryo-TEM (Fröjdh *et al.*, 2022 ▸). With more than 1000 frames per second and a high dynamic range, data collection pipelines need to cope with rates of up to 10 Gb s^−1^ during multi-minute runs for just 512 × 512 pixels. The tiling of these small detectors in the near future implies a quadratic increase in throughput. However, only a small amount of the recorded diffraction patterns will be used for structure determination. Therefore, it is vital to introduce new concepts in data selection to identify the positions of interest.

Here we propose neural networks and traditional ML algorithms for the initial selection of diffraction data. We show that convolutional, fully connected neural networks and support vector machines can successfully generate a model for identifying electron diffraction patterns of amorphous ice, crystalline ice and carbon on holey carbon EM grids.

## 2. Material and methods

### 2.1. Sample preparation

The samples were prepared on a holey carbon coated, 400 mesh copper grid. Grids were loaded with a Tris–HCl buffer solution (20 m*M*, pH 7.5). The samples were frozen using Vitrobot at 90% humidity and 22°C, with blot force 10 and a blotting time of 4 s.

### 2.2. Data collection

#### 2.2.1. EM hardware

The data were collected using a Jeol JEM 2200FS transmission electron microscope with a Gatan 626 cryo-holder. This microscope is equipped with a Schottky field emission gun and a HR (high-resolution) pole piece with a spherical aberration of 1 mm. A Nanomegas DigiSTAR scan generator with custom software was used for orthogonal scanning of the sample in a stepwise mode with a parallel, narrow beam (50–60 nm in diameter), with successive positions being 30 nm apart. The overlap between neighboring exposed areas was therefore approximately 39%. Data were recorded on an ASI Cheetah Medipix3 camera (512 × 512 pixels, sized 55 × 55 µm).

#### 2.2.2. Beam alignment

First, standard alignment for a Jeol JEM 2200FS was executed in ‘TEM mode’ at alpha 2 or 3. (Alpha values are a set of predetermined ratios between the strengths of condenser lenses that control the range of convergence angles on Jeol microscopes. When aligning the microscope for a small parallel beam, the alpha value is set to 1, allowing the beam to remain nearly parallel even at small beam diameter and with a convergence semi-angle of less than 0.1 mrad.) Special care was needed for pivot point alignment (because of the radiation hardness of the Medipix3 detector, this can be performed directly on the detector with a converged beam using a 10 or 30 µm aperture) and alignment of the rotation center using the high-tension (HT) wobbler. To find the correct diffraction plane, the selected-area (SA) aperture was inserted and centered, followed by full spreading of the beam. This caused the SA aperture to select the central, most parallel bundle of the beam. Hereafter, the diffraction focus was set such that the direct beam diameter was minimized and not adjusted further.

To create the smallest semi-parallel beam possible for a Jeol JEM 2200FS, we then switched from ‘TEM mode’ to ‘nanobeam diffraction mode’ (NBD) and spot 1 and alpha 1 were selected for the next step. When switching to NBD mode, the gun and objective lens alignments are retained. The CL1 and mini condenser lenses are fully excited at these NBD settings which leaves only the CL3 lens to be adjusted. To achieve the smallest parallel beam possible, the CL3 lens was adjusted to minimize the direct beam diameter, resulting in a semi-parallel beam with a diameter of 50–60 nm.

In scanning applications for this microscope, a standard dark-field (DF) detector is typically used, which does not record the direct beam. In that case, descan coils are not required to stabilize beam movement, as the higher-angle diffraction signal captured by the DF detector is relatively insensitive to minor fluctuations of the direct beam position. However, we recorded the direct beam and the low-angle diffraction data too, which are more susceptible to minor movements. Because the microscope had no accessible descan coils, minimization of the direct beam position movement at the detector plane during scanning was required due to the long distance between the last crossover of the beam and the camera. The movement could be minimized by optimizing the pivot point alignments. Specifically, extensive refinements of beam tilt pivot points could finally decrease the diffraction pattern movement for each spot by 30%. However, the movement could not be completely neutralized on the experimental system.

#### 2.2.3. Data collection

**Figure 1:**
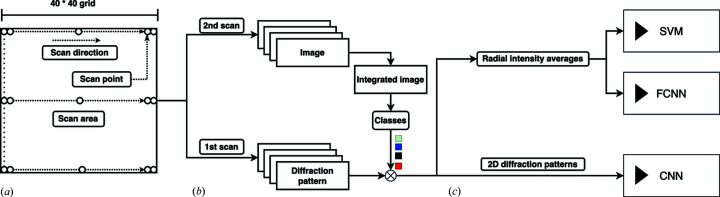
Data collection and processing workflow. (*a*) Data collection from randomly selected scan areas. (*b*) The resulting 1600 diffraction patterns and images from each scan area were gain-corrected, and the integrated image (made from 1600 frame scans in image mode) was used to assign classes to the individual diffraction frames. (*c*) 2D diffraction patterns were used for the convolutional neural network (CNN) and the radial intensity averages for the support vector machine (SVM) and fully connected neural network (FCNN) training.

Where the ice thickness did not obfuscate the carbon edges of the holes in image mode, scan areas were randomly selected and could include ice crystal contamination. Every scan area was scanned twice, once in diffraction mode and once in imaging mode. Immediately following the first scan, the microscope was switched to imaging mode. The image data were required for establishing the classes (carbon, amorphous ice, crystalline ice, mixed) at each scan point. No beam movement was observed for multiple scans across each scan point as switching only involves changing lenses below the sample plane. To prevent significant hysteresis, the lenses were relaxed between the two modes. Earlier experiments demonstrated high reliability in scan points, with deviations less than 1 nm when switching between modes. Images were captured at a magnification that allowed all scan points to be detected on the camera. Each image was recorded in a separate frame, creating two 40 × 40 data sets of the same scan area [Figs. 1 ▸(*a*), 1 ▸(*b*)]. To generate the integrated image, the second data set in image mode was combined by summation.

The virtual camera length was set to 90 cm, corresponding to a resolution of 2.7 Å for detector positions 150 pixels (0.825 mm) from the direct beam. Despite the direct beam moving over the detector during scans, this camera length allowed for the recording of 2.7 Å resolution data in all frames. Camera frames were synchronized with the scanning by the Nanomegas scan software, without engaging beam blank between scan points. With an estimated 2 e^−^ Å^−2^ for a single scan point, the dose rate was approximately 200 e^−^ Å^−2^.

#### 2.2.4. Data pre-processing

In this study, a total of 17 data sets were used, which were obtained from 17 different scan areas. Each data set consisted of a set of 1600 diffraction frames and a set of 1600 corresponding image frames, with each set of frames collected in the same grid of 40 × 40 scan positions. Of the 17 data sets, one was recorded during a different session with very similar, but not necessarily identical alignment parameters.

Individual frames from the data sets were gain-corrected using flat fields and checked for completeness. The image frames were shifted to their inferred location and summed to get an overview of the scanned area [Fig. 1 ▸(*b*)]. We drew region-of-interest (ROI) polygons to select areas labeled as ‘amorphous ice’ and ‘crystalline ice’. To account for uncertainty in areas and overlapping regions where the beam could simultaneously hit multiple area types, ‘mixed’ regions were added on the edges of the selected ROIs. Remaining regions were labeled as ‘carbon’ [Fig. 1 ▸(*b*)].

Subsequently, each individual scan point was assigned one of the four classes described above. The diffraction data, corresponding labels and associated metadata were stored as *MATLAB* structure files.

These steps ensured the data sets were properly prepared and labeled for subsequent analysis and classification tasks.

### 2.3. Pearson cross-correlation (PCC) calculation

The PCC coefficients reveal the linear correlation between the diffraction frames of all scan positions within a data set, with values between −1 and +1 indicating the degree of (anti-) correlation (Knapp & Brown, 2014 ▸). All PCC coefficients were calculated by correlating each diffraction frame with all others from the same data set. The PCC coefficients matrix was constructed by normalizing the covariance between the variables of each individual scan point represented as *X* and *Y* here. The covariance between two observations can be defined according to

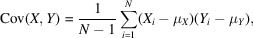

where 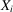 and 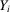 are the *i*th values of the corresponding variables, and 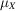 and 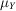 the respective mean values. The 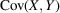 function produces the covariance matrix *C* with the variances on the diagonal which are used for the normalization to obtain the PCC coefficients as

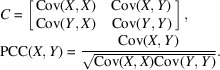




### 2.4. Radial intensity average (RIA) calculation

In the absence of protein, an isotropic diffraction signal is expected for most diffraction frames, because the beam is much wider than the size of the diffracting molecules of the water and carbon regions. To reduce the dimensionality of the data and the impact of noise in the diffraction frames, we calculated their binned radial averages as

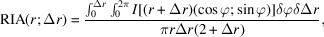

where *r* represents the radial distance from the center 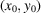 of each diffraction pattern, defined as *r* = 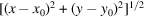 . Δ*r* is the width of the radial range (typically 1 pixel). 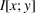 is the measured intensity at a given coordinate.

Because, in its current configuration, our hardware does not keep the central beam at a fixed position of the diffraction frame during scanning, we refined the beam center position of gain-corrected images prior to calculating the RIAs. The diffraction patterns were centered on a 1032 × 1032-pixel image. We used the *Fast 2D peak finder* algorithm (Natan, 2021 ▸) to find the beam center and calculated the offsets in pixels from the set center. A mesh grid was put over the patterns consisting of lattice points at the centers of each pixel. To calculate the RIA, we created 516 bins with a step size of 1-pixel length. These bins spanned from the center to the edge of the images. The intensity values recorded within these bins were summed and averaged, while pixels outside the image frames were discarded. Beam centering is more computationally demanding than calculating the RIA. We are therefore actively working on implementing electron-optical methods to maintain a constant beam center location throughout the scanning process.

### 2.5. Training workflow

We randomly selected 15 data sets for training from the 16 data sets that were all recorded in the same session. The data were split into 90% training and 10% validation parts to avoid overfitting issues. The trained model was tested on the two remaining data sets (3200 diffraction frames) that were excluded from the training. Data set 1 was collected during the same data collection run and data set 2 was recorded at a different time point but with a similar beam alignment. The performance of the classification network was assessed using confusion matrices, where predictions were compared with selected classes obtained from TEM images. The array containing the initial class information and the predicted ones was arranged on a 40 × 40 grid.

#### 2.5.1. Fully connected neural networks

**Figure 2:**
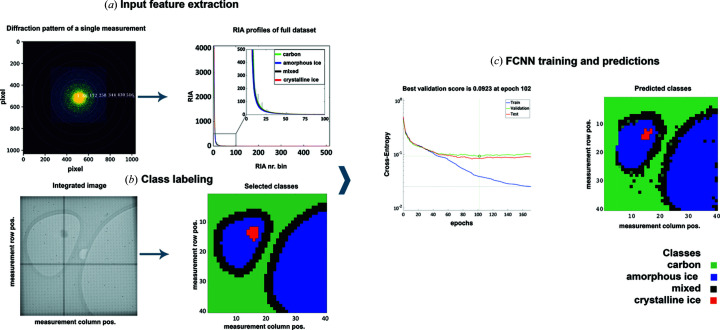
Workflow of the FCNN training and model validation. (*a*) RIA calculations, showing six rings with their radius in pixels, overlaying a diffraction pattern; RIAs plotted against the bin number/pixel radius. (*b*) Integrated image and class representation; a cut-out of the integrated image, incorporating all 1600 scan positions, is shown. The color-coded image represents the classes used for the supervised training procedure. The cross that is visible in these images is a result of the tiling of the Medipix3 detector. (*c*) Supervised training and class predictions of the neural network, colored according to the legend.

We used the RIA as input feature vector to train a fully connected neural network (FCNN), implemented by the *MATLAB* pattern recognition framework (Morton *et al.*, 2014 ▸) (Fig. 2 ▸). The hiddenSizes argument was set to 30, the training function was Conjugate Gradient with Powell/Beale Restarts, and the loss function calculation was based on Categorical Cross-Entropy. The FCNNs have been extensively investigated, are universal approximators (Hornik *et al.*, 1989 ▸) and can be very fast. However, they may not be as discriminative as more sophisticated architectures that we also explored (see Section 2.5.3).

#### 2.5.2. Support vector machines

The same RIAs that were used for the FCNN training were imported into the support vector machine (SVM) classifier. SVMs create a maximum-margin hyperplane that splits different classes and maximizes the distance to the nearest cleanly split examples (Shmilovici, 2005 ▸). We used the libsvm-based SVM implementation from the *scikit* library (Pedregosa *et al.*, 2011 ▸) and hyperparameter tuning with RandomizedSearchCV.

#### 2.5.3. Convolutional neural networks

The presence of proteins in the sample induces anisotropic diffraction data, which will affect the RIAs. To address this, we explored the effectiveness of convolutional neural networks (CNNs) that analyze the unaveraged, 2D diffraction frames for distinguishing between carbon and water regions, which may contain proteins. CNNs are typically constructed with three fundamental components: convolution, pooling and fully connected layers (Cun *et al.*, 1989 ▸). The first two types perform feature extraction, whereas the fully connected layers map the extracted feature vector to the final output (Yamashita *et al.*, 2018 ▸).

**Figure 3:**
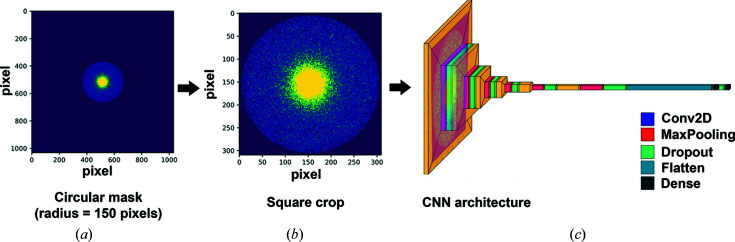
Schematic illustration of the data processing and CNN training: (*a*), (*b*) pre-processing of the diffraction data, (*c*) the architecture of the trained CNN.

Due to movement of the position of the direct beam over the detector during scanning, the data distribution for higher scattering angles was not uniform. To mitigate this, we limited the resolution to 2.7 Å, which encompasses the primary rings of amorphous carbon and amorphous ice. These rings correspond to the first *d*-spacing values of approximately 2–4 Å for amorphous carbon and the water ring at 3.7 Å of amorphous ice [Figs. 3 ▸(*a*), 3 ▸(*b*)]. Consequently, we selected a region with a radius of 150 pixels from the diffraction patterns to capture the relevant information. The implementation of the CNN was carried out using the Keras library with the *TensorFlow* backend (Abadi *et al.*, 2016 ▸).

### 2.6. Performance metrics

We assessed our results using metrics that are common in ML. These metrics are calculated from the confusion matrix, which provides a comprehensive summary of how well a model predicts the classes in a data set. It is a square matrix, where each row corresponds to an actual class and each column corresponds to a predicted class. The values of the matrix indicate how often a certain class is predicted for an instance of an actual class. From the confusion matrix, we can calculate the following numbers:

*True positives* (TP) are correctly predicted classes and are found on the diagonal of the confusion matrix.

*False positives* (FP) are instances that are wrongly predicted as belonging to a particular class. To calculate the FP for a specific class, we sum up all the instances predicted as that class (a column in the confusion matrix) and then subtract the true positives for that class.

*False negatives* (FN) are instances that are wrongly predicted as belonging to other classes. To calculate the FN for a specific class, we sum up all the instances of that class (a row in the confusion matrix) and then subtract the true positives for that class.

From these numbers, we can calculate the following metrics for each of the classes, and below we report their average over all classes:

The *Precision* measures the proportion of true positives out of all instances predicted as positive:

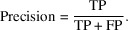




The *Recall* measures the ability of a classification model to identify all positive instances correctly:

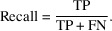




The *Accuracy* measures the overall correctness of a classification model:

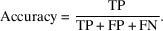




The *F1 score* considers both the ability to avoid false positives (precision) and the ability to find all positive instances (recall):

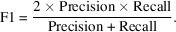




## 3. Results

### 3.1. PCC coefficients and RIA profile

**Figure 4:**
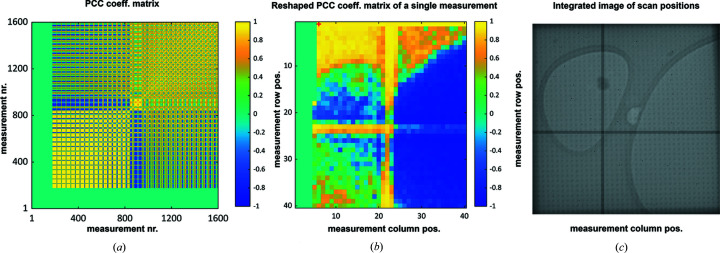
PCC coefficient matrix. (*a*) PCC matrix displaying the correlation between scan points within a data set, ranging from a perfect positive correlation in yellow to a perfect anti-correlation in blue. Entries up to the 177th position are missing in this data set. (*b*) A single slice of the full PCC matrix was reshaped into a 40 × 40 grid to represent the linear relationship of the scan point at position [6; 1] (marked with a small red cross), with the rest of the data set. (*c*) Integrated image of all scan points showing the amorphous ice and the holey carbon support in lighter gray surrounded by the carbon in a darker gray tone. The large cross visible in all panels is caused by the tiling of the Medipix3 detector.

The PCC calculation resulted in a 1600 × 1600 matrix of values between −1 and 1 (Fig. 4 ▸). By slicing and rearranging this PCC coefficient matrix into multiple 40 × 40 matrices, the linear correlations of individual scan points were visualized (Fig. 4 ▸). These indicated a reasonable internal correlation between diffraction data of solvent regions, of carbon support regions and of crystalline ice.

**Figure 5:**
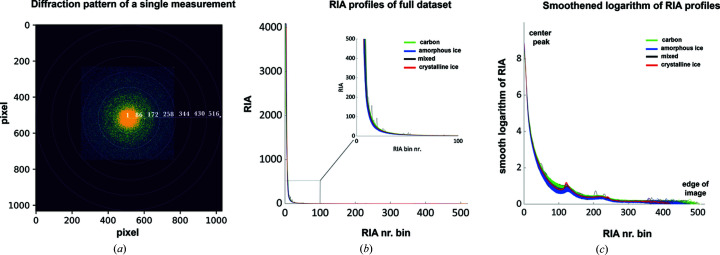
Single diffraction frame and RIAs for different classes. (*a*) Pixel selection for RIA calculations, overlaying a diffraction pattern of a single measurement. (*b*) RIA plots with each curve colored according to the corresponding class. A section is magnified to highlight the clustering of curves depending on the sample. (*c*) Same plot with the smoothened logarithm of the RIA to highlight the differences between classes. To reduce the impact of noise and outliers in the visual representation, the data are shown as a moving average of logarithm (RIA) over 13 bins.

The amorphous ice and carbon regions on holey carbon grids showed diffraction patterns with diffuse rings characteristic of amorphous samples, that were clearly visible in the RIAs [Fig. 5 ▸(*a*)]. The RIAs therefore potentially enable fast training times due to the reduced number of input features (516 input parameters for each measurement, instead of 512 × 512 pixel values).

### 3.2. Training results

#### 3.2.1. Fully connected neural networks

**Figure 6:**
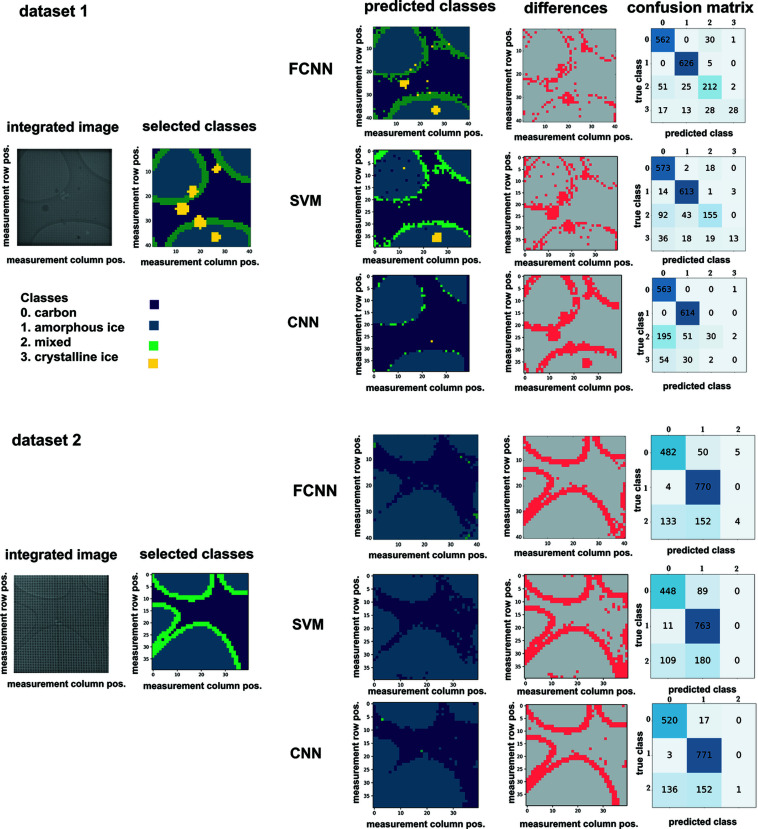
The performance of the FCNN, SVM and CNN was tested on two data sets. Data set 1 was collected during the same data collection run as the training data sets, while data set 2 was acquired at a different time point but with a similar beam alignment. Integrated images of scan points, selected classes, predictions and differences (red) are shown for each approach. The final column represents the confusion matrices.

The FCNN could reach 89.25% accuracy and a weighted F1 score of 88.3% for the test data set collected during the same data collection period (Fig. 6 ▸, data set 1). Most of the misclassifications were related to the ‘mixed’ class, corresponding to the overlap of the amorphous ice and carbon classes around the edges of the holes and edges around crystalline ice. The performance of the FCNN was sensitive to small deviations in alignment and environmental factors, as shown by the drop in accuracy when we used data collected in a different session (Fig. 6 ▸, data set 2). The differentiation between the amorphous ice and carbon was still possible to some degree. Again, most of the misclassifications corresponded to the ‘mixed’ cases. The network reached 78.5% accuracy and a weighted F1 score of 71.1% for this data set (Table 1 ▸).

We concluded that a relatively straightforward FCNN was able to recognize the two main classes when trained on radial profiles of diffraction patterns.

#### 3.2.2. Support vector machines

For the SVM training, the best result was achieved using the Radial Basis Function (RBF) kernel. The SVM classifier could reach 84.6% accuracy and a weighted F1 score of 82% for data set 1. The values were 76% and 68%, respectively, for data set 2 (Table 1 ▸). The iteration through hyperparameters by RandomizedSearchCV did not improve the results. The SVMs were also able to generalize for a data set collected at a different time point (Fig. 6 ▸, data set 2).

#### 3.2.3. Convolutional neural networks

Our CNN architecture consisted of six convolutional layers followed by maximum pooling and dropout layers [Fig. 3 ▸(*c*)]. We used Adam as the first-order gradient-based optimization algorithm and accuracy as a performance metric (Kingma & Ba, 2014 ▸). We chose validation loss as an early stopping metric; the training usually stopped after 100 epochs.

As in the FCNN and SVM cases, the CNNs misclassified some of the ‘mixed’ classes, reaching an overall accuracy of 79% and 81% for data sets 1 and 2, respectively (Table 1 ▸). The weighted F1 score was 71% and 73%, respectively.

The CNN architecture, even when using the central part of diffraction data (up to a resolution of 2.7 Å), was able to perform equally well for the test data set collected at different time points (Fig. 6 ▸, data set 2). However, it was not able to efficiently distinguish crystalline ice from other classes. A likely explanation of the failure to identify ice crystals is that we only included information up to the first ice ring for the CNN and the ice crystals may not have diffracted Bragg spots within the resolution limit used for classification by the CNN. For the purpose of separating vitreous ice from carbon support, the performance metrics of the CNN were highest among other models when tested on a new data set (Table 1 ▸, data set 2).

#### 3.2.4. Speed and performance comparison

The same hardware was used to assess the difference in the prediction speeds. Our implementation of the radial average calculation is computationally demanding and requires 97 s for 1600 diffraction frames (averaged over three trials). The pre-processing for the CNN classification does not require RIAs and is much faster. The FCNNs are the fastest for pre-processed data predictions, whereas SVMs require up to 10 s. CNNs seem more robust, as they have better prediction metrics for the data set that was collected during a different session (Table 1 ▸, data set 2).

## 4. Discussion and conclusions

Single-molecule electron diffraction is an alternative, general method for structural studies of dynamic biomolecular complexes and, with the introduction of modern hybrid pixel detectors, has the potential to further boost the advancement of the field. However, single-molecule electron diffraction uses a narrow electron beam and stepwise scanning mode, which requires a high frame rate to collect sufficient amounts of data, which may stretch the data collection pipelines. To reasonably keep up with data production rates, on-the-fly data selection should be considered.

We demonstrate that ML approaches can efficiently and successfully separate regions of carbon from vitreous ice on EM grids, based on narrow, parallel beam electron diffraction data. Both the FCNN and SVM architectures distinguish amorphous ice from carbon on the holey EM grid with high accuracy and can be used for the selection of positions of interest during the data collection procedure. The FCNN is faster due to its simplicity and has a comparable generalization score. We anticipate that, in the presence of protein, the radial average of a diffraction frame is likely to retain sufficient signal of the water or carbon region to ensure a meaningful distinction can be made. If the beam center is fixed and known, calculating the radial average only requires a single pass through the data, and can be incorporated into any data compression algorithm at very little cost. Therefore, we propose employing these approaches as a pre-selection tool for saving storage space by only storing data from the regions predicted as amorphous ice.

The CNN approach distinguished the main classes using only the small, central part of the diffraction frame. This may hamper its ability to identify tiny ice crystals, but it nevertheless is the most robust and fastest approach for 2D diffraction frame classification of non-crystalline patches (see Section 3.2.3). The pre-processing is faster and further tuning may potentially improve the prediction speed. Most of the ‘mixed’ cases were distributed among ‘amorphous ice’ and ‘carbon’ classes and we could reach a classification accuracy of 81% for the test data set 2.

Thus far the models have been solely trained using data sets that were collected using similar electron beam alignment settings. Tests (not shown here) indicated that the performance of our models dropped significantly for more convergent beams. To further improve performance, the heterogeneity of the training data should be increased, by providing data sets with varying settings. While all three tested approaches were capable of correctly identifying amorphous ice diffraction, the CNN approach showed better generalization performance in the task. The CNN classification achieved higher accuracy when applied to data set 2, which was acquired at a different time point and with very similar, but not necessarily identical beam alignment. As an ideal classifier for this type of data must be able to deal with day-to-day variations in microscope conditions without retraining, we therefore consider the CNN approach using non-radially averaged pixel data to be the most promising for future development. Also, since diffraction data of single protein molecules are anisotropic, using diffraction data that are not radially averaged may be critical for separating single-molecule protein diffraction data from vitreous ice, which is the aim of our future investigations.

## Figures and Tables

**Table:** The weighted average precision, recall, accuracy and F1 scores are shown for the two test data sets. NA, not applicable.

Speed comparison	FCNN	SVM	CNN
1a RIA calculation time for the data set (1600 diffraction frames)	97 s	97 s	NA
1b ROI selection and dimensionality match (1600 diffraction frames)	NA	NA	3.24 s
2 Actual prediction time	0.017 s	9.92 s	1.06 s

**Table:** 

Performance metrics (weighted average)	Data set 1	Data set 2	Data set 1	Data set 2	Data set 1	Data set 2
Precision	0.89	0.72	0.84	0.62	0.77	0.84
Recall	0.89	0.79	0.85	0.76	0.79	0.81
Accuracy	0.89	0.79	0.85	0.76	0.79	0.81
F1 score	0.88	0.711	0.82	0.68	0.71	0.73
